# Extracranial Vertebral Artery Aneurysm With Neurofibromatosis Type 1: The Effectiveness of Endovascular Intervention

**DOI:** 10.7759/cureus.74465

**Published:** 2024-11-25

**Authors:** Yuta Fujiwara, Mizuki Kambara, Kentaro Hayashi

**Affiliations:** 1 Neurosurgery, Shimane University Hospital, Izumo, JPN

**Keywords:** cerebrovascular diseases, endovascular interventions, extracranial vertebral artery, neurofibromatosis type 1 (nf-1), vertebral artery aneurysm

## Abstract

Neurofibromatosis type 1 (NF-1) is associated with vascular complications, including stenosis or the occlusion of the abdominal aorta and renal arteries. However, reports on the occurrence of extracranial vertebral artery aneurysms are scarce. A man in his 40s had back pain and was feeling unwell. Contrast-enhanced computed tomography revealed a right hemothorax and aneurysms involving the right thyrocervical trunk and at the right vertebral arterial origin. The patient was successfully treated with endovascular embolization and achieved a full recovery. Notably, extracranial vertebral artery aneurysms tend to be detected at a later age compared to other complications such as optic gliomas of NF-1, and endovascular treatment has proven effective for managing these aneurysms.

## Introduction

Neurofibromatosis type 1 (NF-1) is an autosomal dominant inherited disorder primarily characterized by café-au-lait spots and neurofibromas. It is caused by a genetic abnormality of the 17th chromosome with a prevalence of approximately one in 3,000-5,000 individuals [1]. While intracranial lesions, such as optic gliomas, are well-documented in NF-1, NF-1-associated vascular complications are rare. Most reports of NF-1 vascular complications are stenosis or the development of an abdominal aortic or renal arterial aneurysm, with extracranial vertebral artery involvement being notably less common. Only 29 cases of extracranial vertebral artery aneurysms of NF-1 have been reported; however, a number of reported deaths may be attributed to such vascular complications [2].

Herein, we report the characteristics of an NF-1-associated extracranial vertebral artery aneurysm, which was discovered following the presentation of hemothorax and was treated endovascularly, resulting in a favorable outcome.

## Case presentation

**Figure 1:**
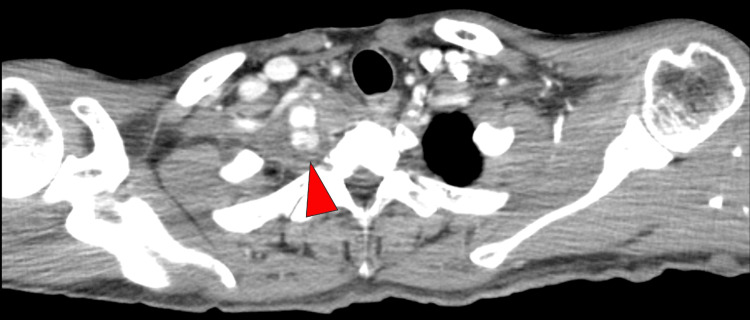
Hemothorax and contrast leakage Contrast-enhanced computed tomography scan. The right hemothorax and contrast leakage near the right thyrocervical trunk (arrowhead)

A man in his 40s with no medical or family history visited our hospital with complaints of back pain and feeling unwell. Contrast-enhanced computed tomography revealed a right hemothorax and aneurysms involving the right thyrocervical trunk and at the origin of the right vertebral artery. Contrast leakage was observed near the thyrocervical trunk, suggesting the potential site of the hemorrhage (Figure 1).

**Figure 2:**
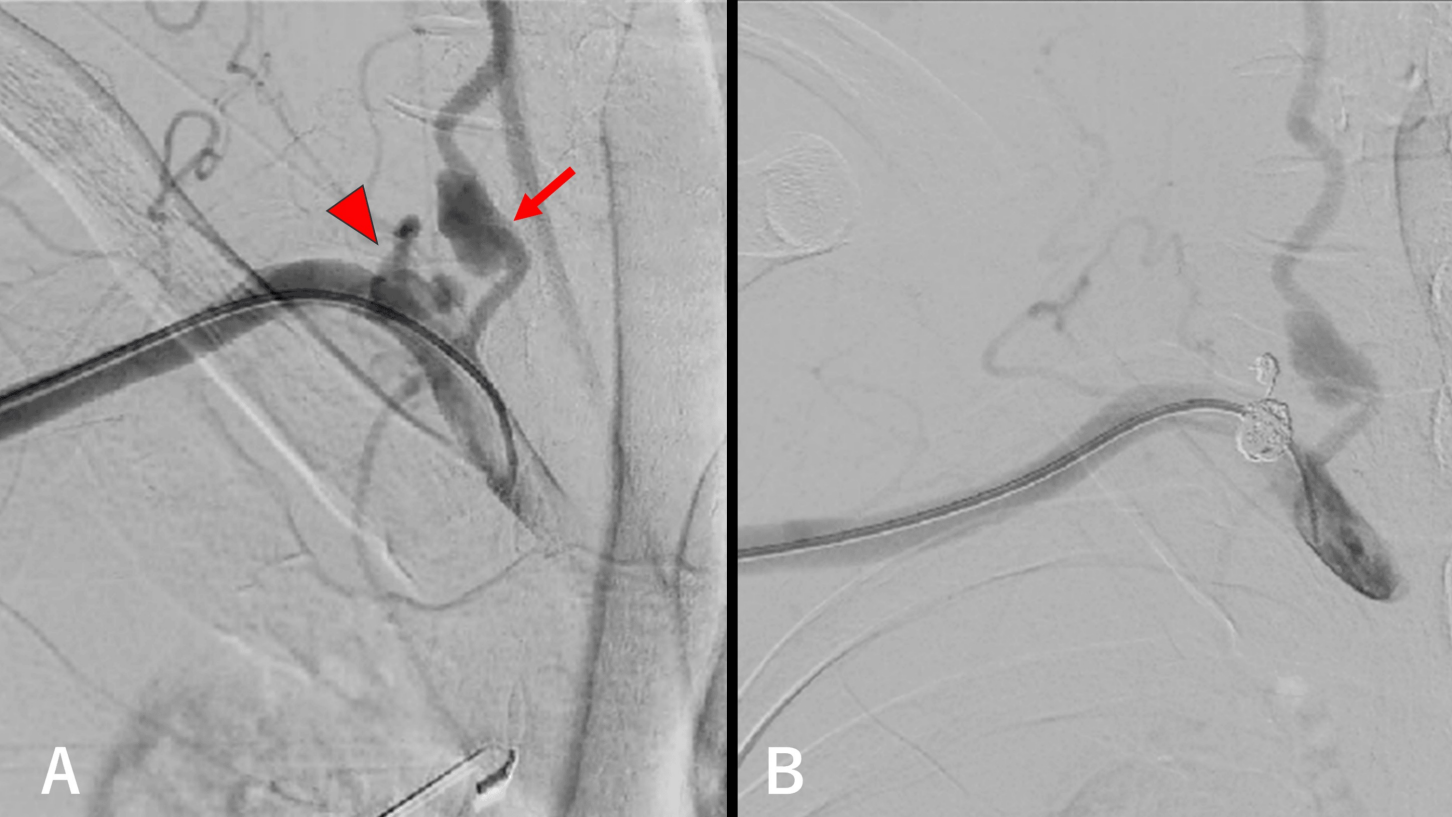
Coil embolization of the right thyrocervical trunk (A) Image of the transbrachial approach. The aneurysm was found in the right thyrocervical trunk (arrowhead) and right vertebral artery at its origin (arrow). (B) The aneurysm in the right thyrocervical trunk was treated with coil embolization

After thoracic drainage, coil embolization was performed (Figure 2A, 2B). The presence of neurofibromas and café-au-lait spots confirmed the diagnosis of NF-1. The general condition of the patient improved, and parent arterial occlusion was performed for the right vertebral artery aneurysm.

**Figure 3:**
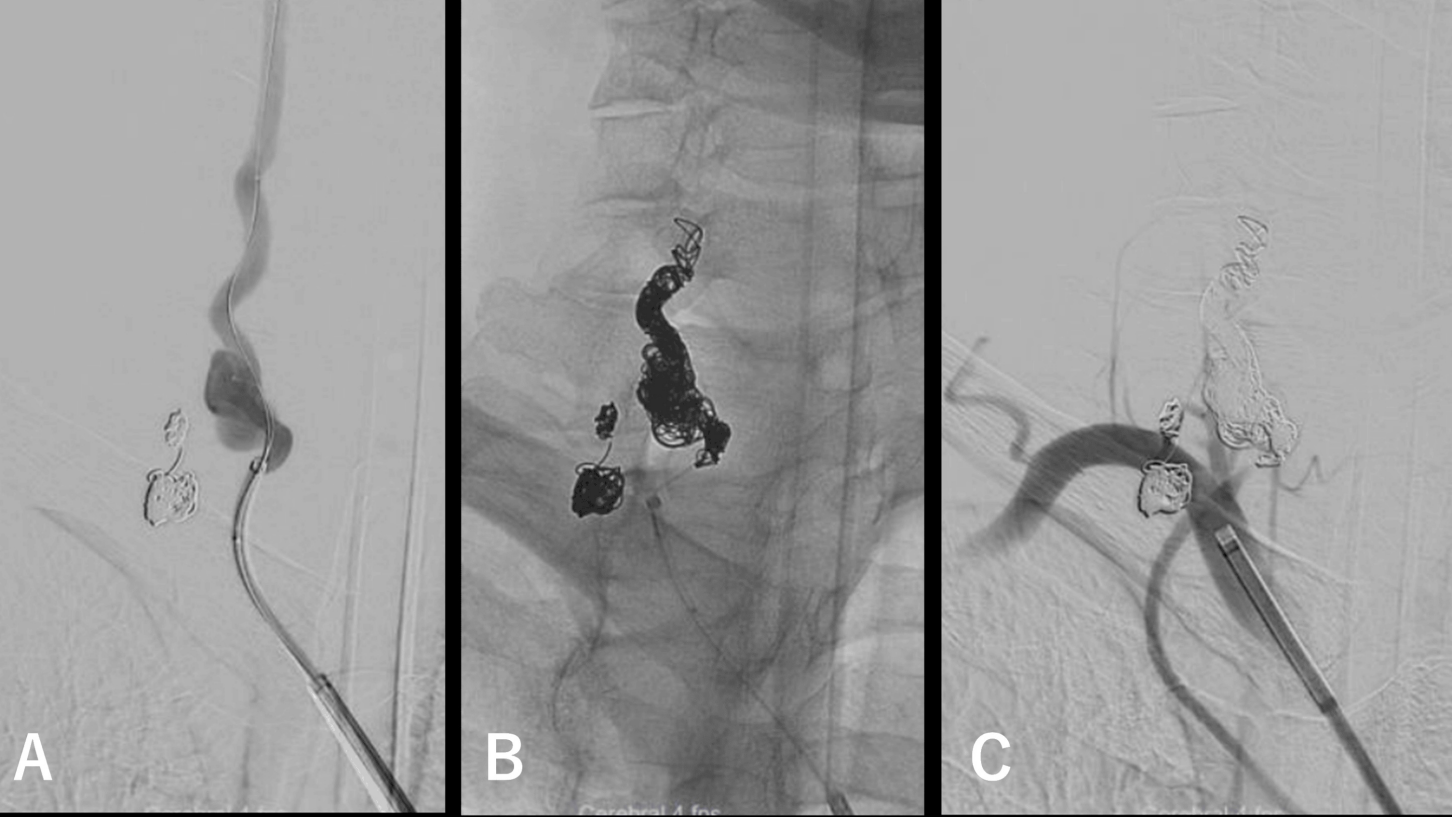
Coil embolization of the extracranial vertebral artery aneurysm (A) A 6 Fr FUBUKI guiding sheath was placed in the brachiocephalic artery, while a 6 Fr Navien was guided to the vertebral artery origin; an SL-10 was placed distal to the aneurysm, while a Headway 17 was placed in the aneurysm. (B) Vertebral artery occlusion was performed using Target XL 360 soft and HydroSoft 3D coils. (C) No contrast leakage was observed in the aneurysm

Under general anesthesia, a 6 Fr FUBUKI guiding sheath (Asahi Intecc, Seto, Japan) was guided into the brachiocephalic artery and a 6 Fr Navien distal access catheter (Medtronic, Minneapolis, MN) was placed at the origin of the vertebral artery. An SL-10 microcatheter (Stryker, Fremont, CA) was guided distal to the aneurysm using a Syncro SELECT standard microwire (Stryker), while a Headway 17 microcatheter (Terumo, Tokyo, Japan) was placed in the aneurysm (Figure 3A). Embolization was performed from within the aneurysm to the proximal portion of the aneurysm using Target XL 360 soft (Stryker) and HydroSoft 3D (Terumo) coils. Subsequently, the embolization of the parent artery was performed from SL-10 distal to the aneurysm using similar coils (Figure 3B). Bilateral vertebral arterial imaging confirmed the absence of any residual aneurysmal dilation (Figure 3C).

The patient's postoperative neurological status was normal, and he was discharged without complications. No recurrence was observed at the three-month follow-up.

## Discussion

NF-1 is an autosomal dominant inherited disorder characterized by café-au-lait spots and neurofibromas, with vascular lesions reported in only 0.4%-6.4% of affected patients [3]. To date, 29 cases of NF-1-associated extracranial vertebral artery aneurysms have been reported, including the present case [2,3]. The median age at diagnosis was 37 years, with 11 cases occurring in men and 19 in women, suggesting a potential sex predilection. Aneurysms occurred on the left side in 22 cases and ruptured in 15 cases. While gliomas, such as optic gliomas, commonly present during childhood, extracranial vertebral artery aneurysms are typically detected in middle age, underscoring the importance of careful follow-up in this population. Although routine screening is not universally recommended due to the rarity of these aneurysms, underdiagnosis remains a concern. Therefore, targeted screening in middle age may be justified in certain cases [2].

Regarding treatment, surgical treatment is recommended even in asymptomatic cases owing to the risk of arteriovenous fistula formation and aneurysm rupture, which is associated with high mortality rates [1]. Two types of hemorrhage have been reported: aneurysm rupture and spontaneous arterial rupture without aneurysm formation [4]. The following mechanisms have been proposed involving the weakening of the vessel's wall: (i) neurofibroma proliferation in the tunica media, (ii) ischemia due to neurofibroma-mediated vasa vasorum compression, and (iii) spindle cell proliferation in the vessel intima causing the thinning of the tunica media and the weakening of the elastic plate [5,6]. Accordingly, endovascular treatment is recommended as open surgery carries a high risk of bleeding [7-9]. Endovascular treatment has been frequently utilized in previous studies. In the present case, two aneurysms were identified in the thyrocervical trunk and the origin of the vertebral artery. Given the suspected vascular fragility, endovascular treatment was performed, yielding favorable outcomes. However, as aneurysms can be induced by catheter-related intimal damage, careful manipulation is necessary. In this case, the parent artery was occluded, but as in past reports, a flow diverter or stenting and coil embolization may have been effective because of the possibility of aneurysmal formation on the contralateral side.

## Conclusions

We report a case of successful endovascular treatment for an extracranial vertebral artery aneurysm of NF-1. Vascular complications of NF-1 require attention. Notably, extracranial vertebral artery aneurysms are typically detected at an older age compared to other complications, such as optic gliomas.

As an aneurysm rupture can be fatal, thus, treatment is recommended. Open surgery can cause excessive bleeding. Endovascular treatment highlights effectiveness in situations involving vascular fragility; however, it requires meticulous handling to avoid intimal damage caused by the catheter.
